# Clinical blindness in conjunction with childhood bacterial meningitis

**DOI:** 10.1038/s41598-023-41685-2

**Published:** 2023-09-19

**Authors:** Tuula Pelkonen, Markku Kallio, Terho Latvala, Irmeli Roine, Heikki Peltola

**Affiliations:** 1 Pediatrics, University of Helsinki and Helsinki University Hospital, Stenbäckinkatu 9, 00029 Helsinki, Finland; 2 New Children’s Hospital, Pediatric Research Center, P.O. Box 347, 00029 HUS Helsinki, Finland; 3 Hospital Pediátrico David Bernardino, Luanda, Angola; 4 Ophthalmology, Helsinki University Hospital, and University of Helsinki, Helsinki, Finland; 5 Faculty of Medicine, University Diego Portales, Santiago, Chile

**Keywords:** Diseases, Medical research, Neurology

## Abstract

Although rarely reported, bilateral loss of vision is a severe complication of childhood bacterial meningitis. We assessed its frequency in five prospective treatment trials performed in Europe, Latin America, and Angola in 1984–2017. Course of illness, follow-up findings, and child’s sight were recorded. Sight was examined at discharge, and conditions permitting, also at 1–3 months post-hospitalization and in Angola on hospital day 7. Experienced pediatricians diagnosed clinical blindness if the child did not make eye contact, did not blink or move the eyes, or remained unresponsive to bright light or movement of large objects before their eyes. Of 1515 patients, 351, 654, and 510 were from Finland, Latin America, and Angola, respectively. At discharge, blindness was observed in 0 (0%), 8 (1.2%), and 51 (10%) children, respectively. In Angola, 64 children appeared to be blind on day 7; 16 of these children died. Blindness found at discharge in Angola was not invariably irreversible; approximately 40% had restored the sight at follow-up visit. Clinical blindness rarely occurred in isolation and was usually associated with young age and poor general condition at hospital arrival. Various other serious sequelae were common among the survivors with clinical blindness.

## Introduction

Severely impaired visual capacity up to bilateral full loss of vision is among the known sequelae of bacterial meningitis (BM) of childhood. This usually occurs in conjunction with other complications, such as hemiparesis, quadriplegia, or mental retardation^1,2^. In a relevant ophthalmological investigation performed in Finland already in the 1950s^3^, 100 cases of BM were compared to 100 “serous” (viral) meningitides. Eye involvement was observed in 23% and 12% percent of cases, respectively; case fatality rates were 22% and 8%, respectively. Ophthalmological signs were associated with disease severity; 83% of children unconscious at presentation exhibited eye symptoms. Tuberculous meningitis, which is known to cause eye problems at approximately twice the rate of other bacterial meningitides^4–6^, was not examined.

Most reports on visual loss after BM are case reports^7–9^. Visual loss was reported from some studies in a review of BM sequelae from 40 studies of African children^10^. Five of these studies reported visual loss at discharge in 1–8% of children^10^. Three studies reported visual loss at a follow-up visit in 0–11% of children^10^.

Our group performed large BM treatment studies in children on three continents^11–15^; visual capacity was also recorded in these studies. Here, we describe our prospectively collected data on this little-known complication of childhood BM.

## Methods

### Patients and data collection

Our five BM trials conducted in 1984–2017 in Finland^11,12^, six countries of Latin America (LatAm)^13^, and Angola^14,15^ have been detailed elsewhere^16^. The study protocols were accepted by the Ethics Committees. Children aged 2 months to 15 years with suspicion of BM were enrolled only after consent from a legal guardian. Trial registration became a practice only during the Angolan studies that were included in the International Standard Randomized Controlled Trial Number Register (number ISRCTN62824827) and in ClinicalTrials.gov (identifier NCT 01540838).

BM was diagnosed if the patient had positive cerebrospinal fluid culture, positive antigen test, or polymerase chain reaction or positive blood culture with compatible symptoms and signs and at least two of the laboratory indices^13–16^ suggesting BM. Children with probable tuberculous meningitis were excluded from the present analysis.

At admission, an attending pediatrician investigated the child thoroughly, performed the spinal tap, and ordered relevant sampling. The pediatrician also completed the specifically designed questionnaire, which was similar in all trials and thus ensured uniform data collection across dissimilar conditions. The child’s presenting condition was graded with the age-adjusted Glasgow Coma Scale (GCS, range 3–15)^17^. This grading was important, as scoring below 13 on GCS in BM indicates increased risk for poor outcomes regardless of etiology^18^. The course of illness was monitored with several clinical and laboratory indices, some of which are shown in Table 1.

**Table 1:** Characteristics of Angolan children admitted with bacterial meningitis and with or without blindness at discharge.

Characteristic	All	Blind at discharge	Not blind at discharge	*p* value
N (%)	510	51 (10)	459 (90)	
Female sex	242/510 (47)	27/51 (53)	215/459 (47)	0.41
Age, years	1.1 (0.5–3.7)	0.7 (0.4–1.3)	1.3 (0.5–3.8)	0.0004
Ill before admission, days	4 (3–7)	7 (3–14)	4 (3–7)	0.0002
Causative bacteria	454	45 (10)	409 (90)	0.047
*Streptococcus pneumoniae*	193/454 (42.5)	24/45 (53.3)	169/409 (41.3)	
*Haemophilus influenzae*	140/454 (30.8)	15/45 (33.3)	125/409 (30.6)	
*Neisseria meningitidis*	77/454 (17.0)	1/45 (2.2)	76/409 (18.6)	
Other bacteria	44/454 (9.7)	5/45 (11.1)	39/409 (9.5)	
At admission				
Glasgow Coma Score	13 (10–15)	10 (7–12)	14 (10–15)	< 0.0001
Seizures before or at admission	214/506 (42)	38/51 (75)	176/455 (39)	< 0.0001
Focal neurological signs at admission^a^	31/500 (6)	8/51 (16)	23/449 (5)	0.003
CSF^b^ leucocytes, /mm^3^	1300(370–3200)	1400 (280–3700)	1300 (377–3000)	0.79
CSF glucose, mg/dL	12.6 (6.0–25.4)	9.9 (6.9–24.0)	13.0 (6.2–25.5)	0.33
CSF protein, mg/dL	189 (113–254)	271 (147–303)	180 (112–251)	0.17
Severe or moderate anemia	451/502 (90)	50/51 (98)	401/451 (89)	0.041
Blood thrombocytes × 10^9^/L	281 (166–442)	365 (196–531)	271 (159–433)	0.010
Malaria thick film positive	127/488 (26)	9/47 (19)	118/441 (27)	0.26
HIV positive	30/419 (7)	3/47 (6)	27/372 (7)	0.83
Sickle-cell disease	30/270 (11)	0/29 (0)	30/241 (12)	0.044
At ward				
Days of fever	3 (2–5)	5 (3–6)	3 (2–5)	0.0008
Days of altered consciousness	2 (0–6)	16 (12–20)	1 (0–5)	< 0.0001
Seizures	268/509 (53)	45/51 (88)	223/458 (49)	< 0.0001
Focal seizures	220/492 (45)	40/50 (80)	180/442 (41)	< 0.0001
Focal neurological signs	131/501 (26)	37/51 (73)	94/450 (21)	< 0.0001
Other focus of infection	299/508 (59)	43/50 (86)	256/458 (56)	< 0.0001
Dehydration	182/508 (36)	26/51 (51)	156/457 (34)	0.017
Treatment				
Supplementary oxygen	213/510 (42)	39/51 (76)	174/459 (38)	< 0.0001
Anti-convulsive treatment	270/510 (53)	45/51 (88)	225/459 (49)	< 0.0001
≥ 2 anticonvulsives vs 1 anticonvulsive	134/270 (50)	33/45 (73)	101/225 (45)	0.0005
Quinine	171/510 (34)	21/51 (41)	150/459 (33)	0.22
Secondary antibiotics	218/509 (43)	33/51 (65)	185/458 (40)	0.0009
Discharge and outcome				
Length of hospital stay, days	11 (9–16)	17 (15–24)	11 (9–15)	< 0.0001
Severe neurological sequelae^c^	56/510 (11)	34/51 (67)	22/459 (5)	< 0.0001
Any neurological sequelae^d^	219/510 (43)	48/51 (94)	171/459 (37)	< 0.0001
Number of neurological sequelae	0 (0–1)	2 (1–3)	0 (0–1)	< 0.0001
Deafness	52/465 (11)	12/49 (24)	40/416 (10)	0.002
Any hearing loss	160/314 (51)	30/39 (77)	130/275 (47)	0.0005
Any neurological or hearing sequelae	275/360 (76)	50/51 (98)	225/309 (73)	< 0.0001

Data are presented as no. (%) or median (interquartile range).

^a^Strabismus, ptosis, nervus facialis paresis, monoparesis, hemiparesis.

^b^Cerebrospinal fluid.

^c^Severe psychomotor retardation, quadriplegia, or hydrocephalus needing a shunt.

^d^Severe neurological sequelae and moderate psychomotor retardation, hemiparesis, monoparesis, or ataxia.

Another thorough clinical investigation was performed at discharge. Special attention was given to neurological and audiological outcomes. “Severe neurological sequelae” were defined as blindness, severe psychomotor retardation, quadriplegia, or hydrocephalus requiring a shunt. “Any neurological sequelae” included those with moderate psychomotor retardation, hemiparesis, monoparesis, or ataxia. Hearing was measured by the Brainstem Evoked Response Audiometry (BERA) or conventional audiometry. Deafness was defined as the better ear’s inability to detect sounds ≥ 80 dB.

Different treatment modifications were tested in our original studies^11–15^. As BM outcomes did not improve (with the exception that glycerol reduced severe neurological sequelae in LatAm^13^), our observations on sight were analyzable as an entirety and the findings could be related to various parameters other than treatment. The long timespan of data collection was not expected to skew this information in any direction.

All data obtained from the daily completed questionnaires were collected by computer and analyzed in Finland. This system also allowed assessment of ophthalmologic parameters. In addition to antibiotics, very ill patients sometimes needed other medications, such as sedatives, antipyretics, and anticonvulsants. In particular, quinine for malaria in Angola was investigated due to its potential influence on sight (and hearing)^19^.

### Definition of clinical blindness

Since ophthalmologist services were virtually non-existent except in Finland, the children’s visual ability was measured by clinical observations. Experienced pediatricians examined sight at discharge, and 1–3 months later if conditions allowed and in Angola on hospital day 7. Clinical blindness was diagnosed if the child did not make eye contact, did not blink or move the eyes, or did not react in response to bright light or movement of large objects before their eyes. Ophthalmoscopy was not performed. A later follow-up visit was planned for all children, but this was not always performed.

### Statistical analysis

All data were computed and analyzed using JMP® Pro 14.1.0 (SAS Institute Inc, Cary, NC, USA) for Windows. Descriptive data are expressed as counts and percentages or medians with interquartile range (IQR), whichever appropriate. Potential differences in the basic characteristics between groups were examined by Wilcoxon/Kruskal–Wallis tests or χ^2^ tests in accordance with data type. We used nominal logistic analysis and calculated odds ratios (OR) with 95% confidence intervals (95% CI) for blindness. For multivariate analysis, we used at-admission-detectable clinical characteristics that in univariate analysis showed *p* value < 0.01.

### Ethics approval and consent to participate

The Luanda Children’s Hospital’s Ethics Committee approved the studies in Angola, and the relevant Ethics Committees or Hospital Boards approved the studies in Latin America and in Finland. Once the registration of clinical trials commenced, the Angolan studies were registered (ISRCTN62824827, 4/10/2005 and NCT01540838, 29/2/2012). The patients were enrolled after written or oral informed consent was obtained from the guardian. All methods were carried out in accordance with the Declaration of Helsinki.

## Results

A total of 1515 children with BM were included; 351 were from Finland, 654 from LatAm, and 510 from Angola. At discharge from hospital, 0 (Finland), 8 (LatAm; 1.2%) and 51 (Angola; 10%) children were blind. Although the large size of the Angolan series was considered, blindness overwhelmingly affected children from this country; approximately 90% of cases (51/59) were from Luanda.

**Figure 1:**
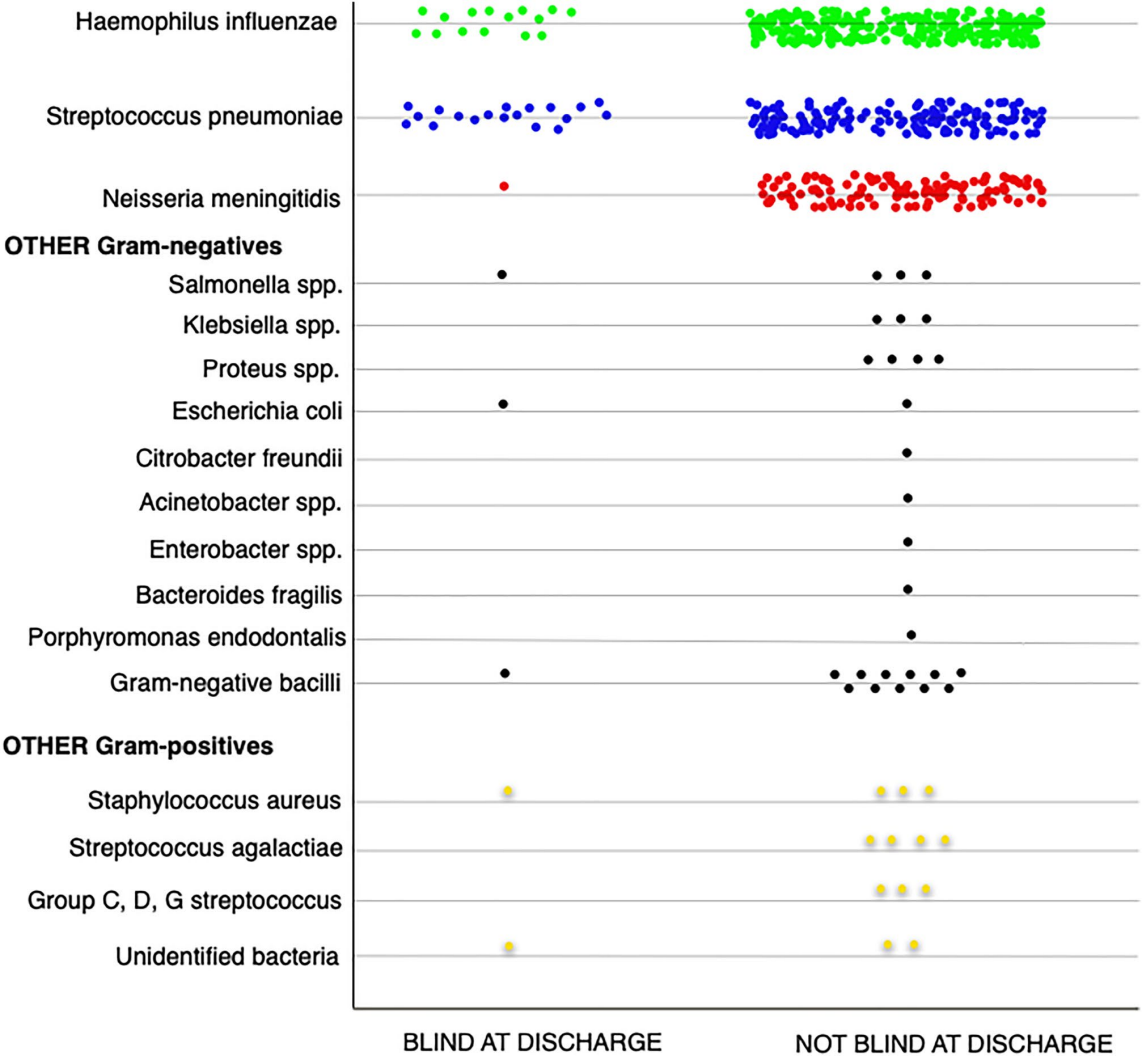
Etiology of bacterial meningitis among the clinically blind and not-blind children, in Finland, Latin America and Angola.

Blindness was associated with causative agent. Vision loss occurred in 25/365 (7%), 18/601(3%), and 1/272 (0.4%) percent with *Streptococcus pneumoniae, Haemophilus influenzae*, and *Neisseria meningitidis* meningitis, respectively (Fig. 1). For the other agents, mostly Gram-negative bacteria, clinical blindness was diagnosed in 6/93(6%). In Latin America, the causative agent was found in 5 of 8 (62.5%) blind children, and was *H.influenzae* in 3, *S.pneumoniae* in 1, and unidentified Gram-positive cocci in 1. In Angola, the causative agent was identified in 44 of 51 (86.3%) blind children, and was *S.pneumoniae* in 24, *H.influenzae* in 15, *N.meningitis* in 1, *E.coli* in 1, *Salmonella* spp. in 1, unidentified Gram-negative bacillus in 1, and *S.aureus* in 1.

The association of blindness with other patient characteristics was examined in Angolan children (Table 1). Blindness was associated with age; children < 1 year were especially vulnerable (odds ratio [OR] 2.57, 95% confidence interval [CI] 1.39–4.73; *p* = 0.002). Delay > 5 days in seeking treatment increased the risk of blindness (OR 2.42, 95% CI 1.35–4.36; *p* = 0.003).

**Figure 2:**
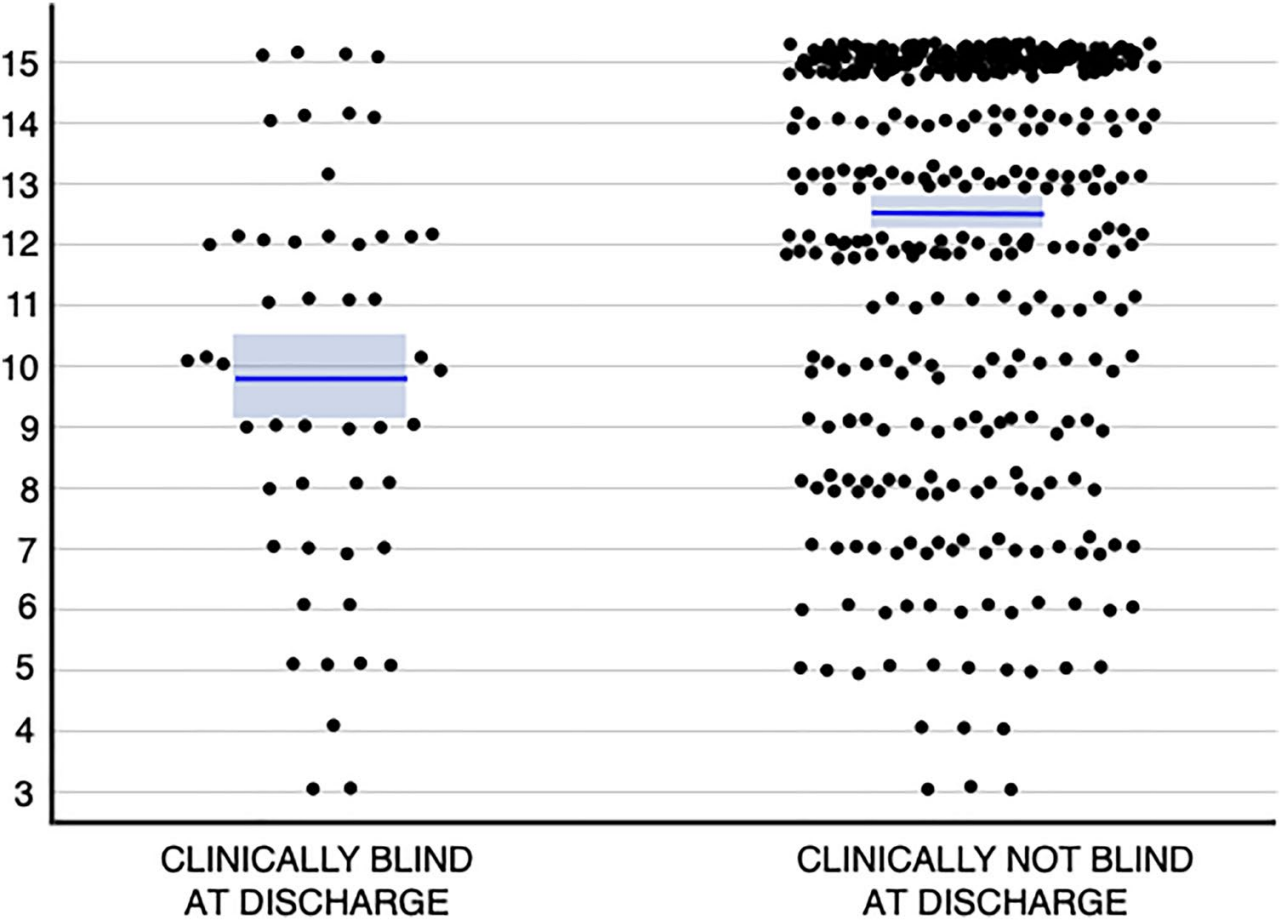
Glasgow Coma Score at admission among the clinically blind and not-blind children in Angola.

Impaired consciousness at hospital arrival was another factor for blindness (Fig. 2). Children with blindness more often had GCS < 13 (42/51; 82.4%) than children without blindness (180/454; 39.6%) (*P* < 0.0001). When compared to children without blindness, children with blindness more often exhibited symptoms and signs indicative of poor outcome, such as seizures before or at presentation, severe anemia, focal neurological signs, or combinations thereof. In contrast, there was no association between blindness and severe underweight, HIV, malaria thick film positivity, or quinine treatment for malaria. Sickle-cell anemia did not increase the risk for blindness.

Table 2 shows multivariate analysis of at-admission detected factors that associated with clinical blindness in Angolan children at discharge. Independent predictors of blindness were GCS < 13, delay > 5 days, age < 1 year, and seizures.

**Table 2:** Multivariate analysis of factors associated with at-discharge clinical blindness of Angolan children with bacterial meningitis.

Characteristic	Odds ratio (95% confidence intervals)	*p* value
Age < 1 year	1.94 (1.01–3.72)	0.046
Ill > 5 days before admission	2.12 (1.13–3.99)	0.020
Glasgow Coma Score < 13 at admission	4.95 (2.23–10.96)	< 0.0001
Seizures before or at admission	2.29 (1.12–4.67)	0.023
Focal neurological signs at admission	1.38 (0.71–2.69)	0.34

Of the patient characteristics manifesting at the ward, seizures, especially focal seizures, other focus of infection beside meningitis, and dehydration were associated with blindness at discharge. Focal neurological signs were found in 37/51 (73%) children with blindness and 94/450 (21%) (*p* < 0.0001) children without blindness. The focal neurological signs of children with blindness were strabismus (n = 22), ptosis (n = 6), monoparesis (n = 4), hemiparesis (n = 3), and nervus facialis paresis (n = 2). Children with blindness experienced fever (5 vs. 3 days; *p* = 0.0008) and altered consciousness (16 vs. 1 days; *p* < 0.0001) longer than children without blindness. Children with blindness also required supplementary oxygen (76% vs. 38%, *p* < 0.0001) and anti-convulsants (88% vs. 49%, *p* < 0.0001) more often than children without blindness.

Blindness was associated with other sequelae, such as severe psychomotor retardation, (30/51 [59%] *vs.* 11/459 [2%]; *p* < 0.0001), quadriplegia, (16/51 [31%] *vs.* 1/459 [0.2%]; *p* < 0.0001), hydrocephalus (9/51 [18%] *vs*. 11/459 [2%]; *p* < 0.0001), and deafness (12/49 [24%] *vs.* 40/416 [10%], *p* = 0.002). Children with blindness were also hospitalized longer (17 vs. 11 days, *p* < 0.0001). Only one child who was diagnosed with blindness did not have other sequelae at discharge. This 20-month-old child had group B meningococcal meningitis and otitis media and regained vision when examined at follow-up visit.

**Figure 3:**
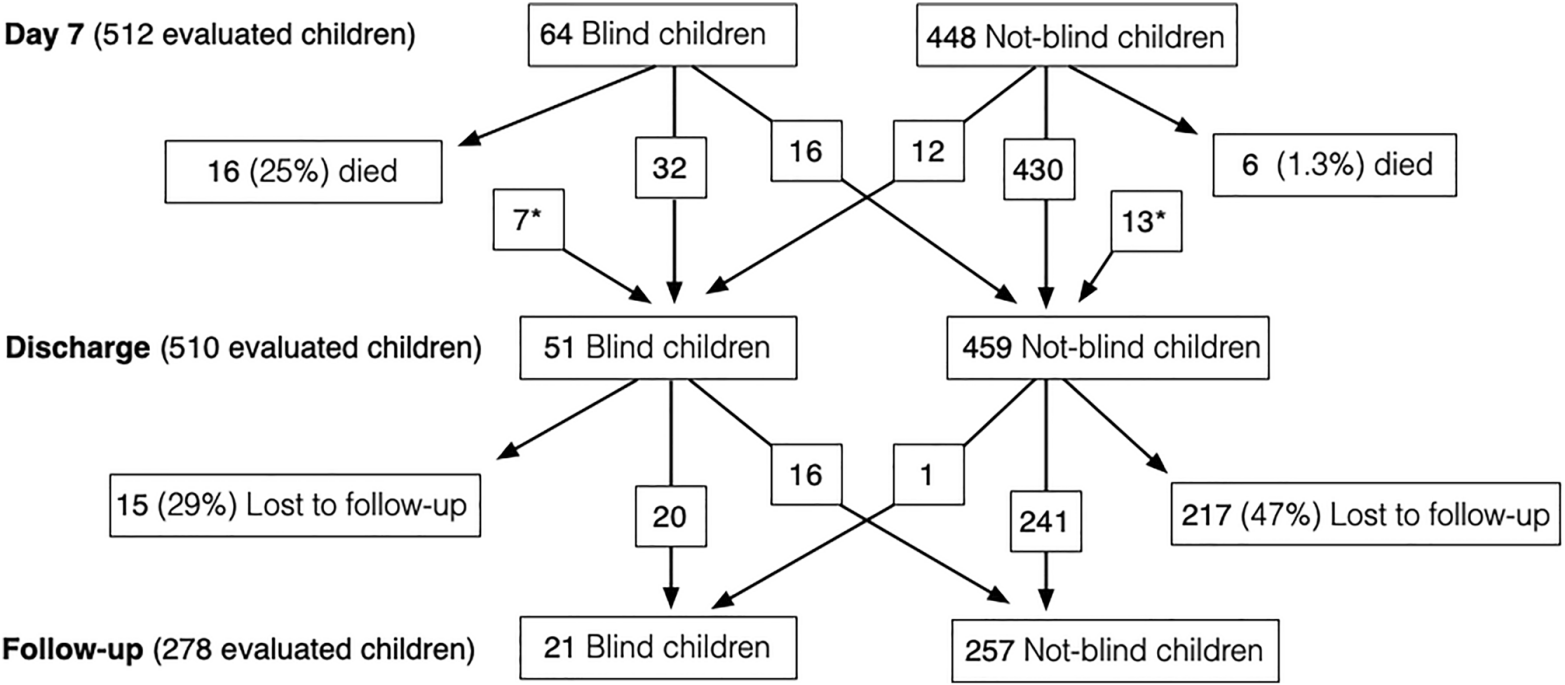
Flow-chart of Angolan children with bacterial meningitis and clinical blindness on day 7, at discharge and at follow-up visit. *Children whose sight was not evaluated on day 7.

Vision was examined in 512 children on day 7, of whom 64 (12.5%) were clinically blind. Of these, 16 (25%) died in hospital (Fig. 3). Only 6/448 (1.3%) of children without visual loss on day 7 died. The children diagnosed with blindness on day 7 were not invariably the same individuals who had blindness at discharge, and vice versa (Fig. 3)*.* Of the 48 surviving children who had blindness on day 7, 16 (33%) were not diagnosed with blindness at discharge.

A follow-up visit (mostly at 1–3 months posthospitalization) was attended by 278 (55%) children, of whom 36 (13%) were diagnosed with blindness at discharge (Fig. 3). The differences of the characteristics of clinically blind vs not blind children at follow-up visit were alike the differences at discharge (Supplementary table 1). In multivariate analysis, only GCS < 13 at admission associated significantly with clinical blindness at follow-up visit (Supplementary table 2). Compared to the situation at discharge, 16 (44%) individuals were no longer clinically blind. When compared with children who were still blind at this follow-up visit, at least four characteristics were associated with this observation: duration of altered consciousness (1 day *vs.* 16 days; *p* < 0.0001), seizures at ward (49% *vs.* 88%; *p* < 0.0001), days with focal neurological signs (3 days *vs.* 13 days; *p* = 0.004), and ataxia (81% *vs.* 100%; *p* = 0.039). Duration of prehospital symptoms, GCS < 13, and severe psychomotor retardation were not significantly associated. A 12-month-old child who had pneumococcal meningitis and was not diagnosed blind at discharge was clinically blind at the follow-up visit.

## Discussion

Despite the considerable impact of vision loss, little is known of blindness in conjunction with BM^1–3,7–10^. This may be explained by the paucity of this complication in industrialized countries; there were no cases identified among the 351 Finnish patients examined. In LatAm, 8/654 children had blindness, whereas in Angola, 51/510 (10%) children had blindness at discharge. The frequency of blindness at discharge in Luanda is similar to the reported 8% in children with BM in South Africa^20^.

Dissimilar etiology and the special risk in *S. pneumoniae* meningitis partly explained the preponderance of blindness in Luanda. Here, blindness at discharge due to pneumococcal, *Haemophilus*, and meningococcal meningitis was observed in 7%, 3%, and 0.4%, respectively. In a previous African review, the corresponding figures were 2%, 1 to 3% percent, and 3%^10^.

Distinguishing BM from cerebral malaria is a concern in tropical Africa. Adjunct quinine was sometimes administered in Luanda. Quinine binds to α1-acid glycoprotein and other plasma proteins; this binding is increased in *P. falciparum* malaria^19^. Therefore, cinchonism, a constellation comprising various symptoms, including visual (and hearing) disturbances up to full blindness, may explain why many children with blindness at discharge regained vision a few months later.

The pathogenesis of vision loss in BM is not precisely known. Anoxia or inflammation of the cerebral cortex, thrombosis, and cerebral infarcts may lead to cortical blindness^1,7,8^. In our study, quadriplegia and focal neurological signs (such as monoparesis and hemiparesis) were more common in children with blindness, suggesting anoxic or circulatory (or both) brain damage. Hydrocephalus was also associated with blindness; this was also observed in tuberculous meningitis, where increased intracranial pressure can cause papilledema and optic neuropathy^5^. Both tuberculous and other bacterial meningitis can cause optic nerve, chiasm, or tractus involvement and damage^5,9^.

In African children with BM, visual loss has been reported at discharge in 1 to 8% and in 0 to 11% at follow-up visit^10^. In a follow-up study from industrialized countries, of 14 children with cortical blindness and mostly *Haemophilus* meningitis, 7 children regained vision, 4 children recovered some vision, and 3 children remained blind^7^. In Bangladesh, of 51 children with pneumococcal meningitis, vision loss was diagnosed in 4 children at 1 month after discharge and in 2 children at 6–24 months after discharge^21^. Spontaneous improvement has also been observed in impaired hearing in conjunction with BM^22^.

This study has some limitations. Ophthalmologic services were not available in Angola and the attending pediatricians did not record pupillary reflexes or eye movements and did not perform ophthalmoscopy. However, it is highly unlikely that a child who is completely unresponsive to light and stimuli (such as moving fingers or toys before their face) would have any meaningful visual capacity. Importantly, examining the vision of children with impaired consciousness or neurological sequelae is also challenging in industrialized countries. Therefore, we believe our information is relevant and sufficiently important to warrant full ophthalmological exploration in future meningitis studies.

Our three series (Finland, LatAm, Africa) were dissimilar in terms of socioeconomic conditions and in many other aspects. However, the disease (BM) was the same, the causative agents were similar, data were collected prospectively in a similar manner, and the entire series is the largest to date. Although the risk of BM in childhood is reduced due to vaccinations, the importance of vision loss associated with this disease merits further attention. We recommend that at least children with BM and risk factors for blindness undergo ophthalmological examinations 1–3 months after hospitalization.

### Supplementary Information

Supplementary Tables.

## Data Availability

The data used and analyzed during the current study are available from the corresponding author upon a reasonable request.
