# Supplementary material for: Clinical blindness in conjunction with childhood bacterial meningitis
Source: Sci Rep. 2023 Sep 19;13:15530. doi: 10.1038/s41598-023-41685-2 (PMC10509259; doi:10.1038/s41598-023-41685-2)
Supplement: Supplementary file 1 — Supplementary Tables. [file 41598_2023_41685_MOESM1_ESM.docx]

| Characteristic | All | Blind at follow-up visit | Not blind at follow-up visit | *p* value |
| --- | --- | --- | --- | --- |
| N (%) | 278 | 21 (7.6%) | 257 (92.4%) |  |
| Female sex | 137/278 (49) | 15/21 (71) | 122/257 (47) | 0.035 |
| Age, years |  | 0.7 (0.35 – 1.0) | 1.3 (0.5 – 3.8) | 0.003 |
| Ill before admission, days |  | 6 (3 – 15) | 4 (3 – 7) | 0.013 |
| Causative bacteria | 253 | 20 | 233 | 0.25 |
| *Streptococcus pneumoniae* | 113 (45) | 9 (45) | 104 (45) |  |
| *Haemophilus influenzae* | 82 (32) | 9 (45) | 73 (31) |  |
| *Neisseria meningitidis* | 35 (14) | 0 (0) | 35 (15) |  |
| Other bacteria | 23 (9) | 2 (10) | 21 (9) |  |
| AT ADMISSION |  |  |  |  |
| Glasgow Coma Score |  | 10 (7 – 12) | 14 (10 – 15) | <0.0001 |
| Seizures before or at admission | 114/277 (41) | 17/21 (81) | 97/256 (38) | 0.0001 |
| Focal neurological signs at admission^a^ | 56/275 (20) | 9/21 (43) | 47/254 (19) | 0.008 |
| CSF^b^ leucocytes, /mm^3^ |  | 1750 (205 – 4750) | 1184 (350 – 2788) | 0.36 |
| CSF glucose, mg/dL |  | 13 (5 – 25) | 12 (6 – 23) | 1.0 |
| CSF protein, mg/dL |  | 220 (163 – 276) | 148 (100 – 246) | 0.39 |
| Severe or moderate anemia | 251/273 (92) | 21/21 (100) | 230/252 (91) | 0.16 |
| Blood thrombocytes × 10^9^/L |  | 403 (180 – 713) | 281 (174 – 426) | 0.039 |
| Malaria thick film positive | 82/269 (30) | 4/20 (20) | 78/249 (31) | 0.29 |
| HIV positive | 22/245 (9) | 3/19 (16) | 19/226 (8) | 0.28 |
| Sickle-cell disease | 14/156 (9) | 0/10 (0) | 14/146 (10) | 0.30 |
| AT WARD |  |  |  |  |
| Days of fever |  | 5 (4 – 6) | 3 (2 – 6) | 0.088 |
| Days of altered consciousness |  | 17 (13 – 29) | 1 (0 – 4) | <0.0001 |
| Seizures | 147/278 (53) | 21/21 (100) | 126/257 (49) | <0.0001 |
| Focal seizures | 130/275 (47) | 19/21 (90) | 111/254 (44) | 0.0002 |
| Focal neurological signs | 78/274 (28) | 17/21 (81) | 61/253 (24) | <0.0001 |
| Other focus of infection | 164/276 (59) | 17/21 (81) | 147/255 (58) | 0.037 |
| Dehydration | 98/277 (35) | 11/21 (52) | 87/256 (34) | 0.090 |
| TREATMENT |  |  |  |  |
| Supplementary oxygen | 120/278 (43) | 16/21 (76) | 104/257 (40) | 0.002 |
| Anti-convulsive treatment | 148/278 (148) | 20/21 (95) | 128/257 (50) | <0.0001 |
| ≥2 anticonvulsives vs 1 anticonvulsive | 77/148 (52) | 16/20 (80) | 61/128 (48) | 0.007 |
| Quinine | 105/278 (38) | 7/21 (33) | 98/257 (38) | 0.66 |
| Secondary antibiotics | 109/278 (39) | 13/21 (62) | 96/257 (37) | 0.027 |
| DISCHARGE AND OUTCOME |  |  |  |  |
| Length of hospital stay, days |  | 19 (15 – 29) | 10 (9 – 15) | <0.0001 |
| Severe neurological sequelae^c^ | 41/278 (15) | 20/21 (95) | 21/257 (7) | <0.0001 |
| Any neurological sequelae^d^ | 137/278 (49) | 21/21 (100) | 116/257 (45) | <0.0001 |
| Number of neurological sequelae |  | 3 (2 – 3) | 0 (0 – 1) | <0.0001 |
| Deafness | 36/270 (13) | 5/20 (25) | 31/250 (12) | 0.11 |
| Any hearing loss | 100/198 (51) | 10/15 (67) | 90/183 (49) | 0.19 |
| Any neurological or hearing sequelae | 173/222 (78) | 21/21 (100) | 152/201 (76) | 0.010 |

**Supplementary Table 1**. Characteristics of Angolan children admitted with bacterial meningitis and with or without blindness at follow-up visit. Data are presented as no. (%) or median (interquartile range). ^a^ Strabismus, ptosis, nervus facialis paresis, monoparesis, hemiparesis. ^b^ Cerebrospinal fluid. ^c^ Severe psychomotor retardation, quadriplegia, or hydrocephalus needing a shunt. ^d^ Severe neurological sequelae and moderate psychomotor retardation, hemiparesis, monoparesis, or ataxia.

| Characteristic | Odds ratio (95% confidence intervals) | *p* value |
| --- | --- | --- |
| Age < 1 year | 1.62 (0.58 – 4.55) | 0.36 |
| Ill > 5 days before admission | 2.22 (0.81 – 6.05) | 0.12 |
| Glasgow Coma Score <13 at admission | 8.91 (1.90 – 41.75) | 0.006 |
| Seizures before or at admission | 2.93 (0.88 – 9.74) | 0.08 |
| Focal neurological signs at admission | 2.25 (0.83 – 6.10) | 0.11 |

**Supplementary Table 2**. Multivariate analysis of factors associated with at-follow-up visit diagnosed clinical blindness of Angolan children with bacterial meningitis.
